# Ultrasound-Responsive Materials for Drug/Gene Delivery

**DOI:** 10.3389/fphar.2019.01650

**Published:** 2020-01-31

**Authors:** Xiaowen Cai, Yuan Jiang, Mei Lin, Jiyong Zhang, Huanhuan Guo, Fanwen Yang, Wingnang Leung, Chuanshan Xu

**Affiliations:** ^1^ Key Laboratory of Molecular Target and Clinical Pharmacology, State Key Laboratory of Respiratory Disease, School of Pharmaceutical Sciences & Fifth Affiliated Hospital, Guangzhou Medical University, Guangzhou, China; ^2^ Department of Pediatrics, Shenzhen Maternity and Child Health Care Hospital, Shenzhen, China; ^3^ Department of Biomedical Engineering, School of Basic Medical Sciences, Guangzhou Medical University, Guangzhou, China; ^4^ Asia-Pacific Institute of Aging Studies, Lingnan University, Tuen Mun, Hong Kong, Hong Kong

**Keywords:** ultrasound-responsive materials, drug, gene, delivery, microbubbles

## Abstract

Ultrasound is one of the most commonly used methods in the diagnosis and therapy of diseases due to its safety, deep penetration into tissue, and non-invasive nature. In the drug/gene delivery systems, ultrasound shows many advantages in terms of site-specific delivery and spatial release control of drugs/genes and attracts increasing attention. Microbubbles are the most well-known ultrasound-responsive delivery materials. Recently, nanobubbles, droplets, micelles, and nanoliposomes have been developed as novel carriers in this field. Herein, we review advances of novel ultrasound-responsive materials (nanobubbles, droplets, micelles and nanoliposomes) and discuss the challenges of ultrasound-responsive materials in delivery systems to boost the development of ultrasound-responsive materials as delivery carriers.

## Introduction

Drugs are important agents for combating the ailments. Drugs are mainly divided into hydrophilic and lipophilic types according to solubility. Hydrophilic drugs, in general, have difficulties entering cells through passive diffusion because cell membranes are composed mainly of lipid bilayers (Thansandote et al., 2015). However, lipophilic drugs are often difficult to dissolve in water and have unsatisfactory bioavailability (Arnott and Planey, 2012). Recently, gene drugs including DNA drugs, RNA drugs have shown promise in treating mutant gene-associated diseases (Kaufmann et al., 2013). Different from chemical drugs, these gene drugs are much larger and have difficulties entering cells. Meanwhile, gene drugs are easily degraded by nucleases in blood stream or cells.

To address the shortcomings of chemical and gene drugs in clinical practices, drug-delivery carriers are used to encapsulate drugs to improve the water solubility of lipophilic drugs, enhance the penetration of hydrophilic drugs into cells, and decrease the side-effect of drugs. For example, the nanoliposomal encapsulation improve the water solubility and bioavailability of hydrophobic polyphenol curcumin (diferuloylmethane) and enhance anticancer activity of curcumin against breast cancer (Hasan et al., 2014). Additionally, delivery systems can also protect gene drugs from degradation by extracellular and intracellular enzymes, and promote therapeutic outcome (Cavalieri et al., 2015).

Advanced drug delivery systems (DDS) require a demand of dosage, spatial, and temporal control strategy (Liu et al., 2016b). Several studies have shown that microspheres and nanoparticles can protect drugs or genes and further improve therapeutic outcomes (Nakamura and Harashima, 2017; Alkie et al., 2019; Holley et al., 2019; Yu et al., 2019). However, the uncontrolled release of drugs and genes at the disease site is the main limitation of microspheres and nanoparticles.

Since 1978, stimuli-responsive delivery systems have been widely investigated to control release of drugs and genes in targeted sites (Yatvin et al., 1978). Recently, the commonly used stimuli include microenvironment pH and enzymes in target tissues, as well as external stimuli such as photons, electromagnetic, and ultrasound waves. It supplies new perspective for the study of control release of drugs and genes in delivery system. Ultrasound wave is a promising physical stimulus for drug/gene delivery because of its safety, low cost, and portability of ultrasound instrument (Endo-Takahashi et al., 2013).

Ultrasound, including low frequency (<100 kHz) and high frequency (>100 kHz and MHz range) ultrasound (Ji et al., 2018; Matafonova and Batoev, 2019), as one of the most commonly used physical factors has been widely employed in the disease diagnosis and therapy (Witte et al., 2018). Since the mid-1990s, it has been demonstrated that ultrasound can enhance the permeability of agents into living cells (Lentacker et al., 2014). Ultrasound sonication improves the delivery efficiency of drugs/genes mainly through thermal and non-thermal effect (Husseini and Pitt, 2008a; Lentacker et al., 2010; De Temmerman et al., 2011; He et al., 2015; Tardoski et al., 2015; Endo-Takahashi et al., 2016; Liao et al., 2017). The thermal effects are produced from the absorption of acoustic energy in biological tissues. While the non-thermal effects are mainly generated from ultrasound pressure, acoustic streaming, shockwaves, liquid microjet, and ultrasound-induced oscillation or cavitation (Marin et al., 2002; Husseini and Pitt, 2008a; Mannaris et al., 2020). In particular, in the presence of cavitation nuclei, a type of particles which can lower acoustic intensity to induce cavitation, ultrasound shows higher delivery efficiency (Miller et al., 1999; Ward et al., 1999; Peruzzi et al., 2018; Mannaris et al., 2020).

In view of the advantages of cavitation nuclei in ultrasound stimuli, microbubbles as cavitation nuclei have been used widely in ultrasound-mediated drug/gene delivery (Huang et al., 2012; Yan et al., 2015; Oishi et al., 2016; Wang et al., 2016; Zullino et al., 2018). The commonly used microbubbles have gaseous cores and outer shells composed of phospholipids, polymers or proteins. The size of microbubbles (about 1–10 μm) enables them to circulate with red blood cells (Jayaweera et al., 1994; Sirsi and Borden, 2012; Mulvana et al., 2017). Microbubbles, as proven ultrasound-responsive materials, have been applied in drug delivery in clinical trials (**Table 1**) (Hynynen et al., 2001; Dimcevski et al., 2016; He et al., 2016). These clinical trials confirmed the controllability of delivering the cargo like drugs and gene materials with ultrasonic switch and visualization of treatment. Most noteworthy, many preclinical studies were also under study. Kuo et al. (2019) used doxorubicin-loaded microbubbles in combination with ultrasound (1 MHz) to facilitate the entering of doxorubicin into osteosarcoma cells and exhibited 3.7-fold inhibition of cancer growth compared to doxrubicin-loaded microbubbles without sonication, and simultaneously in combination with contrast-enhanced ultrasound imaging doxorubicin-loaded microbubbles were used to monitor the perfusion and volume of cancer. Lee et al. (2016) delivered miR-29b-3p to enhance fracture healing using ultrasound microbubbles system. Even in articular cartilage to which it is difficult to deliver drugs, ultrasound-responsive microbubbles can also improve the drug delivery efficiency (Nieminen et al., 2017). However, microbubbles have a short circulation time in blood because their sizes restrict their passage through the barrier between blood vessels and targeted tissues. For example, tumor tissues permit only smaller particles (<1 μm) to enter their interior (Zullino et al., 2018). In particular, nanoparticles of size 1–100 nm can have high accumulation in tumor tissues *via* the enhanced permeability and retention (EPR) effect (Maeda, 2001; Baghbani and Moztarzadeh, 2017).

**Table 1:** Clinical trials of materials assisting drug delivery under sonication [the datasets for this table can be found in the (ClinicalTrials.gov) (https://clinicaltrials.gov/)].

Materials	NCT number	Cargo	Center frequency	Therapeutic area
Microbubbles	NCT 03458975	Monoclonal antibodies in combination with chemotherapy	Not Provided	Colorectal Cancer, Hepatic Metastases
Microbubbles	NCT 03199274	Perflutren Protein-Type A Microspheres	Not Provided	Hepatocellular Carcinoma, Liver Cancer
Microbubbles	NCT 02233205	platinum and gemcitabine	1.9 MHz	Gastrointestinal Neoplasms
Microbubbles	NCT 01674556	Gemzar	1.9 MHz	Pancreatic Adenocarcinoma
Microbubbles	NCT 01678495	Recombinant tissue plasminogen activator	Not Provided	Cerebrovascular Stroke
Liposomes	NCT 03749850	Lyso-thermosensitive liposomal doxorubicin and Cyclophosphamide	Not Provided	Metastatic Breast Cancer, Breast Cancer Breast, Neoplasms, Stage IV Breast Cancer, Metastatic Cancer, Invasive Ductal Carcinoma of Female Breast, Invasive Ductal Breast Cancer, Adenocarcinoma Breast
Liposomes	NCT 02536183	Lyso-thermosensitive liposomal doxorubicin	Not Provided	Pediatric Cancer, Solid Tumors, Rhabdomyosarcoma, Ewing Sarcoma, Soft Tissue Sarcomas, Osteosarcoma, Neuroblastoma Wilms Tumor, Hepatic Tumor, Germ Cell Tumors
Liposomes	NCT 02181075	Lyso-thermosensitive liposomal (LTSL) doxorubicin	0.96 MHz	Liver Tumor

Along with the rapid development of nanomaterials, nanoscale bubbles, droplets, micelles and nanoliposomes have been developed as novel nanomaterials in ultrasound-responsive drug-delivery systems (Ulrich, 2002; Ahmed et al., 2015). Some liposomes have been applied for drug delivery under ultrasound in clinical trials (**Table 1**).

Herein, we will introduce several of the major nanoscale ultrasound-responsive materials used in drug/gene delivery. Furthermore, we will discuss the challenges and the development of ultrasound-responsive materials in drug/gene delivery.

## Novel Ultrasound-Responsive Materials

### Nanobubbles

Nanobubbles are a type of nanoscale bubbles (1–1,000 nm) with gaseous cores and outer shells. As a ultrasound-responsive material, nanobubbles were designed originally as contrast agents to enhance ultrasound imaging, and developed as drug-delivery carriers later (Cavalli et al., 2016).

In tumor tissues, the endothelial gaps range from 380 nm to 780 nm (Hobbs et al., 1998). Microbubbles with the size of 1–10 μm cannot generally extravasate from blood vessels to tumor tissues. However, “leaky” tumor vessels and obstructive lymphatic drainage make nanobubbles with the size of 10–780 nm extravasate through endothelial gaps and accumulate in tumor tissue *via* the EPR effect (Fernandes et al., 2018). Therefore, nanobubbles show great potential in drug/gene delivery for the diagnosis and therapy of cancer because they can accumulate in tumor tissues and interact with tumor cells directly. Upon ultrasound sonication, nanobubble-induced sonoporation on cells can also enhance the efficiency of drug/gene delivery (Xing et al., 2016). As early as 2009, Watanabe et al. (2010) used ultrasound-responsive nanobubbles to control the delivery of gene to skeletal muscle both in BALB/c mice. This is the first report to use isotopic imaging (PET or SPECT) to realize visualization of gene transfection and to provide an easy way to detect the transfection of gene in clinic especially in vascular diseases and muscular dystrophy. Wu et al. (2017a) used poly(lactide-co-glycolic acid) (PLGA) as the shell and octafluoropropane (C_3_F_8_) gas as core of nanobubbles to load paclitaxel, and further modified them with A10-3.2 aptamer to target prostate cell-specific membrane antigen (PSMA) for therapy of prostate cancer. Under low-frequency ultrasound stimuli, the nanobubble (PTX-A10-3.2-PLGA NB) achieved high drug release that induced significant apoptosis *in vitro* and significant inhibition of growth of tumor cells in BALB/c nude mice with xenograft tumors, and provided biological imaging of prostate-cancer cells. Subsequently in 2018, this research team synthesized cationic nanobubbles (CNBs) with same gas core decorated with A10-3.2 aptamer (siFoxM1-Apt-CNBs) for anti-tumor-targeted delivery of siRNA-FoxM1(Forkhead box M1) (Wu et al., 2018a). The transfection efficiency of siRNA was improved significantly, whereas FoxM1 expression was reduced significantly after siFoxM1-Apt-CNBs combined with ultrasound stimuli in xenograft tumors in nude mice as well as in PSMA-positive LNCaP cells *in vivo*. These actions led to significant inhibition of tumor growth and prolonged mice survival.

Cai et al. (2018) used C_3_F_8_ gas as the core and phospholipids as shells to prepare nanobubbles for delivering isocitrate dehydrogenase 1 (IDH1)-siRNA to gliomas. The siRNA-loaded nanobubbles interfered significantly expression of IDH1 *in vitro* and *in vivo* under ultrasound sonication. Shen et al. (2018a) modified ultrasound-mediated resveratrol-embedded nanobubbles containing C_3_F_8_ core with anti-N-cadherin 2 antibody (which is regarded as a specific binding ligand of nucleus pulposus cells in intervertebral disks) to increase the drug concentration in intervertebral disks for slowing down their degeneration *in vivo*. Song et al. (2018b) developed low-frequency ultrasound-responsive nanobubbles composed of C_3_F_8_ core and PEGylated lipid shell to deliver a plasmid, the expression vector of brain-derived neurotrophic factor (BDNF) for treating acute injury to the spinal cord, and microtubule-associated protein 2 (MAP-2) antibody to modify the nanobubbles to enhance the targeting. They found that combined treatment of ultrasound and nanobubbles increased BDNF expression significantly *in vitro* and *in vivo*, and improved recovery of spinal-cord injury, indicating that nanobubbles are potential ultrasound-responsive materials in drug/gene delivery. Some other studies are enumerated in **Table 2**.

**Table 2:** Summaries of the studies on ultrasound-responsive nanobubbles.

Core	Shell	Cargo	Ultrasonic frequency	Therapeutic area	Study
Gas-generating calcium carbonate	PEG-PAsp	Doxorubicin	40 MHz	Squamous Cell Carcinoma	(Min et al., 2015)
C_3_F_8_	Herceptin- PEGylated phospholipid-shell	No cargo	5–12 MHz	Her-2-positive Breast Cancers	(Jiang et al., 2016)
CF_4_	PLGA	Doxorubicin	1 MHz	VX2 Liver Tumor	(Meng et al., 2016)
C_3_F_8_	Herceptin-PEG-PLGA	Paclitaxel	1 MHz and 40 MHz	Breast Cancer	(Song et al., 2017)
C_5_F_12_	Glycine/PEG/RGD- modiﬁed poly(methacrylic acid)	Doxorubicin	Not Provided	Liver Tumor	(Li et al., 2017)
Oxygen	Sodium carboxymethylcellulose	Mitomycin-C	40 MHz	Bladder Cancer	(Bhandari et al., 2018)
C_3_F_8_ + UCNP–CN	DPPC, DSPE-PEG2k and DPPA	Doxorubicin	7 MHz	Tongue Squamous Carcinoma	(Chan et al., 2018)
C_3_F_8_	Mix of DPPC and DPPA	pc DNA3.1(+)/PNP plasmid	1.3 MHz	Hepatocellular Carcinoma	(Zhang et al., 2018a)
C_3_F_8_	Folate-conjugated N-palmitoyl chitosan	No cargo	7 MHz	Oral Epidermoid Cancer Cells, Cervical Cancer, Lung Cancer	(Shen et al., 2018b)
C_3_F_8_	Mix DSPC, DSPE-PEG2000 and DSPE-PEG2000-biotin	Apatinib	1 MHz	Liver Tumor	(Tian et al., 2018)
C_5_H_2_F_10_	Polymer shell composing of chitosan and lecithin	Paclitaxel and survivin inhibitor sepantronium bromide	3 MHz	Lung Cancer	(Baspinar et al., 2019)
C_3_F_8_	PLA-PEG-NH_2_	No cargo	9.0 MHz	Breast Cancer	(Shang et al., 2019)
1% CO_2_	Protein	The pEGFP and pCMV-Luc reporter plasmids	18 MHz	Breast Cancer	(Tayier et al., 2019)

### Droplets

Droplets are especially ultrasound-responsive liquid nanomaterials consisting of volatile perfluorocarbons (PFCs). It can undergo a phase transition through ultrasound-induced acoustic droplet vaporization or heat. After ultrasound stimulation, droplets can expand and convert into nanobubbles. This characteristic feature improves the ultrasonic contrast and triggers the release of loading agents specifically. Moreover droplets are more stable than gas bubbles in blood circulation at 37°C because droplets maintain their liquid core in the circulation avoiding gas dissolution (Lanza and Wickline, 2001; Lea-Banks et al., 2019). Stable PFC emulsions, commonly used droplets, can be prepared to ~200 nm in diameter (Fabiilli et al., 2010), which is beneficial for circulating for a longer time *in vivo*, passing into tissues or cells, and enhancing the EPR effect (Shpak et al., 2016). More interestingly, Lattin et al. (2015) supposed that the disruption of droplets may break down the membrane of endosome to aid the escape from the endosome endocytosis pathway of macromolecules such as genes. Their findings provide a new strategy for delivering therapeutic agents especially large molecules like genes upon ultrasound sonication.

Droplets, in general, are used to load lipophilic drugs, such as 10-hydroxycamptothecin (HCPT). HCPT is an efficacious anticancer drug but has limited clinical application due to its poor hydrophilicity. Encapsulation of lipophilic materials could improve the therapeutic efficacy of HCPT against cancer (Zhang et al., 2008; Li et al., 2012; Yang et al., 2013; Liu et al., 2015; Liu et al., 2016a). Based on this information, Liu et al. (2018) prepared an ultrasound-responsive droplet consisting of four parts: folic acid (FA) for overexpression of FA receptors on cancer cell membranes; superparamagnetic Fe_3_O_4_ for imaging; HCPT for cancer treatment; a PFC as the droplet core. The PFC core could undergo droplet vaporization upon sonication to cause HCPT release and enhance ultrasound imaging.

Rapoport et al. (2011) developed a novel nanoemulsion containing a perfluoro-15-crown-5-ether (PFCE) core with good stability and reversible transition from droplet to bubble. Moreover, the novel nanoemulsions could realize ultrasound and ^19^Flourine magnetic resonance dual-mode imaging, and enhance the inhibitory efficiency of paclitaxel-loaded nanoemulsions on the growth and metastasis of breast and pancreatic cancer cells in mice.

Doplets were also investigated in the application of brain diseases. Chen et al. (2013) compared the safety of microbubbles and droplets for drug delivery to the brain under focused ultrasound. In their studies, the same lipid compositions were used as the outer shells of microbubbles and droplets: perfluorobutane as the microbubble core and PFC as the droplet core. The cavitation induced by droplets required a higher threshold and droplets could deliver the drug more safely and more effectively than microbubbles in the brain. In 2018, another study on the delivery of biomolecules into the brain using droplets was published by colleagues in the team (Wu et al., 2018a). These findings demonstrated that ultrasound droplet-mediated delivery was a novel approach to deliver drug/gene into the brain effectively. Other up-to-date researches are listed in **Table 3**.

**Table 3:** Summaries of the studies on ultrasound-responsive droplets.

Core	Shell	Cargo	Ultrasonic frequency	Therapeutic area	Study
C_6_F_14_	Alginate	Doxorubicin and curcumin	28 k Hz and 1 MHz	Multidrug Resistant Ovarian Cancer	(Baghbani and Moztarzadeh, 2017)
C_5_F_12_	PLGA	Cetuximab and 10-Hydroxycamptothecin	1 MHz	Anaplastic Thyroid Carcinoma	(Wang et al., 2018)
C_9_F_20_	Mix of DSPC and mPEG-DSPE	Lidocaine	2.25 MHz	Acute and Chronic Pain	(Soto et al., 2018)
C_5_F_12_	Perylene diimide	ZnF_16_Pc	40 MHz	Malignant Glioblastoma	(Tang et al., 2018)
C_6_F_14_	Phosphatidyl ethanolamine	Ce6	1 MHz	Breast Cancer	(Yu et al., 2018)
C_3_F_8_	Mix of DSPE-PEG3400-t Ly P-1, DPPG, DPPC, and cholesterol	10-Hydroxycamptothecin	1 MHz	Breast Cancer	(Zhu et al., 2018)
C_5_F_12_	Mix of POPC, POPE, cholesterol, and DSPE-PEG-2000	Camptothecin	2 MHz	Melanoma	(Ho et al., 2018)
C_5_F_12_	Mix of DPPC, DSPE- m PEG2000, cholesterol	IR780	650 k Hz for treatment, 12 MHz for imaging	Breast Cancer	(Zhang et al., 2019)
C_6_F_14_	O-carboxymethyl chitosan	Doxorubicin	9.0 MHz	Prostatic Cancer	(Meng et al., 2019)
C_7_F_16_	Pluronic F68	Basic ﬁbroblast growth factor	2.5 MHz	Ischemic Cardiovascular Diseases	(Dong et al., 2019)
C_6_F_14_	Polydopamine	No cargo	7.5 MHz	Breast Cancer	(Mannaris et al., 2020)

### Micelles

Micelles are, in general, generated through self-assembly of polymers containing a hydrophilic group and a hydrophobic alkane (Husseini et al., 2007). Moreover, the diameters of micelles, which range from 10 nm to 100 nm, will help their application in nanoformulations (Husseini and Pitt, 2008b; Xia et al., 2016). Amphiphilic structures enable hydrophilic drugs and hydrophobic drugs to be encapsulated readily in micelles. The moderate thermal effect induced by ultrasound can increase the cell membrane penetrability resulting in enhancing extravasation in targeted cells (Rapoport, 2012). And increasing evidence has shown that micelles can be destroyed under shockwaves produced by ultrasound to release cargo loaded in micelles and deliver them to target tissues (Ahmed et al., 2015). Ultrasound-responsive micelles not only achieve the control of space release but also the quantity of release, since they can reassemble again when the ultrasound shuts off (Husseini et al., 2002; Tanbour et al., 2016). Hence, micelles are also potential materials for ultrasound-responsive delivery.

As early as in 2006, Chen et al. (2006) prepared micelles composed of three kinds of pluronics, F127, L61 and P85 as gene-delivery carriers under sonication. They found that, upon sonication, these three types of micelles enhanced the efficiency of gene transfection in 3T3-MDEI, C2C12, and CHO cell lines. Later, Wu et al. (2017b) developed a mixed micelle of pluronic P123/F127 polymers to encapsulate curcumin. They showed that curcumin was released at specific sites under ultrasound sonication, and that sonication increased cellular uptake of curcumin compared with that using free curcumin. *In vitro*, curcumin released from micelles increased along with increasing ultrasound intensity. Furthermore, curcumin-loaded micelles decreased the tumor weight by ~6.5-fold upon ultrasound sonication compared with the group without sonication exposure. Kang et al. (2019) studied doxorubicin (DOX) release with the help of high-intensity focused ultrasound (HIFU). The center frequency of the pre-clinical HIFU system they used was 1.5 MHz. Under high-intensity focused ultrasound, the structure of micelles loaded with DOX and hydrophobic 1,3-bis-(2,4,6-trimethylpheny l) imidazolylidene nitric oxide (IMesNO, a donor of nitric oxide, NO) was destroyed, and IMesNO was released from the micelles to produce NO. In cancer tissues, NO improved the EPR effect by expanding cancer blood vessels to increase blood blow, and subsequently enhanced the anticancer effect of DOX.

### Nanoliposomes

Liposomes show excellent biocompatibility because they consist primarily of lipid bilayers (Schroeder et al., 2009). Liposomes can often load hydrophilic molecules and lipophilic molecules to improve their pharmacokinetics and reduce systemic toxicity (Torchilin, 2005; Allen and Cullis, 2013). Recently, accelerating evidence shows that nanoliposomes can deliver and release drugs/genes in target tissues upon ultrasound sonication (Dromi et al., 2007; Mannaris et al., 2013; Ta et al., 2014; Lyon et al., 2017). In general, nanoliposomes do not contain gas, so they are not particularly responsive to ultrasound. To achieve a particular response to ultrasound, nanoliposomes can be designed to contain vapor-phase molecules or encapsulated emulsions that can vaporize under ultrasound (Huang, 2008; Geers et al., 2012). When being exposed to ultrasound, cavitation or thermal effects can increase the release of drug/gene-loaded in nanoliposomes. Usually under sonication at high frequency, thermal effect takes the main role of delivery process. While under low frequency, cavitation plays an important role (Huang and MacDonald, 2004; Kopechek et al., 2008; Smith et al., 2010; Lattin et al., 2012).

To improve the targeting ability of ultrasound-responsive nanoliposomes, Negishi et al. (2013) used an AG73 peptide targeting syndecan (which is highly expressed in neovascular vessels) to modify liposomes with a perfluoropropane core. This AG73 peptide-modification endowed liposomes with a perfluoropropane core to have good targeting ability to tumor cells and deliver plasmids to them effectively. In 2018, a new liposome-encapsulating gas, phosphorodiamidate morpholino oligomer, was used to induce antisense oligonucleotide-mediated “exon skipping” for treating Duchenne muscular dystrophy (Negishi et al., 2018). This new liposome could deliver the antisense oligonucleotide to diseased muscles and release it upon ultrasound sonication.

Nowadays, a mixture of liposomes and microbubbles termed a “liposome–microbubble complex” (LMC) has been reported. The LMC has the high drug-loading ability of liposomes and ultrasound-responsive property of microbubbles. Zhang et al. (2018b) fabricated a LMC as a drug vehicle to deliver paclitaxel. To overcome the disadvantage that LMC was effective *in vitro* but not *in vivo*, they used iRGD peptide, a nine-unit cyclic tumor-homing and tissue-penetrating peptide, to modify the LMC to achieve better permeability into blood vessels and tissues in a tumor-speciﬁc manner. This modified LMC showed higher toxicity to 4T1 breast cancer cells and antitumor efficacy in a subcutaneous tumor model.

## Challenges

Ultrasound-responsive material-based drug/gene delivery has been explored widely in treating cancer (Khokhlova et al., 2015; Qin et al., 2016; Fan et al., 2017; Yue et al., 2018; Jing et al., 2019), cardiovascular diseases (Hua et al., 2014; Dixon et al., 2015; Castle and Feinstein, 2016), orthopedic diseases (Lee et al., 2016; Pullan et al., 2017; Kuo et al., 2019), ocular diseases (Aptel and Lafon, 2012; Wan et al., 2015a; Wan et al., 2015b; Lafond et al., 2017) and brain diseases (Timbie et al., 2015; Song et al., 2018a), and also applied in vaccine immunization (Tachibana et al., 1997; Escoffre et al., 2016). However, application of ultrasound-responsive materials in drug/gene delivery faces certain challenges.

First, the prerequisite for treating diseases is a sufficient amount of drug/gene delivered and released in diseased tissues. Most ultrasound-responsive materials need an ultrasound-responsive core (gaseous, PFC, or gas-generating). These ultrasound-responsive cores consume a lot of space in ultrasound-responsive materials (microbubbles, nanobubbles, or droplets), which makes lower drug/gene-loaded contents, and decrease the amount of drug/gene delivered to diseased tissues, and eventually lead to limited therapeutic efficacy (Klibanov et al., 2010; Fabiilli et al., 2010; Shende and Jain, 2019). Second, nanoscale ultrasound-responsive materials have advantages over microbubbles with regard to targeted delivery of drugs and genes, but these nanomaterials are less responsive than microbubbles (Sirsi and Borden, 2014). So nanomaterials require higher ultrasound intensity to induce cavitation for effective release of drugs/genes from nanomaterials. But ultrasound of high intensity can cause damage to neighboring healthy tissues. High-intensity ultrasound also induces the rapid collapse of bubbles and rapid release of the drug/gene loaded in the bubbles, which may not meet the need for sustained release of some drugs (e.g., insulin).

The last but not the least, ultrasonic parameters are still noticeable issues. Low- and high-frequency ultrasound can damage biologic tissues when sonication-induced heating is too high, and the pore formation on cell membranes is irreversible (Mehier-Humbert et al., 2005). Therefore, the intensity and duration of ultrasound sonication must be controlled. Kovacs et al. (2017) found that pulsed-focused ultrasound induced the opening of the blood–brain barrier and was accompanied by increased expression of heat-shock protein 70, interleukin-1, interleukin-18, tumor necrosis factor-α, and inflammation of brain tissues, suggesting that application of ultrasound-responsive materials in drug/gene delivery to the brain system should be done with extreme caution.

## Conclusions

Ultrasound-responsive materials can deliver drugs/genes to targeted tissues, and induce the release of drugs/genes in specific sites upon ultrasound sonication. However, most evidence has arisen from *in vitro* and *in vivo* animal experiments. Few clinical trials have investigated the role of ultrasound-responsive materials in drug/gene delivery. Thus, more clinical trials should be conducted to confirm the outlook of ultrasound-responsive materials in drug/gene delivery.

Recent studies have revealed that the major reason limiting application of ultrasound-responsive materials is their low drug/gene-loaded content. Enhancing the drug/gene-loaded content in ultrasound-responsive materials will be a “hotspot” for clinical translation of ultrasound-responsive materials.

In addition, sonoporation is regarded to be the main reason that ultrasound-responsive materials enhance the release of loaded drugs/genes. However, the interaction of ultrasound and ultrasound-responsive materials is complicated, and can induce mechanical forces, sonoporation, heating, and sonochemical effects. Therefore, better understanding of how ultrasound-responsive materials enhance release of loaded drugs/genes will lay a solid foundation to boost development of ultrasound-responsive materials in drug/gene delivery.

## Data Availability Statement

The datasets generated for this study can be found in the ClinicalTrials.gov database (https://clinicaltrials.gov/).

## Author Contributions

XC, YJ, and CX contributed to the conception and design of the study. XC wrote the first draft of the manuscript. YJ wrote sections of the manuscript. All authors contributed to manuscript revision and approved the submitted version.

## Funding

This work was supported by the Fund of Talents for High-level University in the Construction of Guangzhou (B195002009025) and the Science and Technology Project of Guangdong Province (2017B090911012).

## Conflict of Interest

The authors declare that the research was conducted in the absence of commercial or financial relationships that could be construed as a potential conflict of interest.
